# Associations Between Type‐Specific Sedentary Behaviour and Low Back Pain: Evidence From a Large‐Scale Cohort Study

**DOI:** 10.1002/msc.70118

**Published:** 2025-05-12

**Authors:** On Lee, DooYong Park

**Affiliations:** ^1^ Department of Sport Science Korea Institute of Sports Science Seoul Korea; ^2^ Department of Physical Education Seoul National University Seoul Korea

**Keywords:** aged, epidemiology, low back pain, sedentary behaviour

## Abstract

**Objective:**

This study aimed to investigate the association between different types of sedentary behaviour and the risk of low back pain (LBP) and LBP intensity.

**Methods:**

Data were obtained from a large‐scale cohort survey of Koreans comprising 2742 participants. Sedentary behaviours—namely, TV viewing (TV‐ST), squatting (Squat‐ST), and floor (Floor‐ST) sitting—were categorised into three groups (< 2 h, 2–4 h, and ≥ 4 h). LBP was evaluated using the Oswestry Disability Index (ODI) and a visual analogue scale (VAS). Multiple logistic regression analyses were performed to calculate odds ratios (OR) and 95% confidence intervals (CI), and multiple linear regression analyses were used to estimate *β* coefficients with 95% CI.

**Results:**

For TV‐ST, after adjusting for all confounding variables, participants watching TV for 2–4 h and ≥ 4 h exhibited LBP risk ORs of 1.23 (OR = 1.23, 95% CI = 1.01–1.50) and 1.44 (OR = 1.44, 95% CI = 1.14–1.82), respectively, compared with those watching TV for less than 2 h. Similar results were observed for Squat‐ST, with an OR of 1.83 (95% CI: 1.33–2.56) for 2–4 h and an OR of 4.14 (95% CI: 1.96–8.72) for ≥ 4 h. Furthermore, distinct associations were observed between sedentary behaviour types and LBP severity as measured by VAS and ODI scores.

**Conclusion:**

These findings highlight the critical role of specific sedentary behaviours in the development and severity of LBP, underscoring the need for targeted interventions in its prevention and management.

## 1 Introduction

Low back pain (LBP) is one of the most commonly experienced musculoskeletal conditions throughout life, particularly among older adults (Tsuji et al. 2021). LBP is known to reduce quality of life (Jonsdottir et al. 2019), and accelerate social retirement (Lötters and Burdorf 2006), making it a significant global public health concern due to its substantial economic burden (Bontrup et al. 2019). According to national statistics on LBP, 28.5% of 50,195 survey respondents reported experiencing LBP at some point, with a particularly high prevalence (51.5%) among individuals aged 60 years and older. Additionally, 73.1% of 14,316 respondents reported experiencing work‐related LBP, indicating a high level of exposure to LBP risk in the general population (Agency, 2023). Previous research has demonstrated a strong association between physical activity, physical function, and LBP in older adults (Cedraschi et al. 2016), While sedentary behaviour was traditionally categorised as ‘physical inactivity’, it is now recognised as a distinct behaviour that includes activities such as driving, screen use, and prolonged sitting while reading or reclining. These behaviours have been identified as risk factors for various health conditions (Tsao et al. 2022). Prolonged sitting time (ST), in particular, has been linked to reduced postural variation and intervertebral disc degeneration, both of which are primary contributors to LBP (Korshøj et al. 2018).

Although many studies have investigated the relationship between LBP and sedentary behaviour, most have not classified different types of sedentary behaviours (Chen et al. 2009; Korshøj et al. 2018), Furthermore, studies utilising accelerometers to measure ST were unable to assess the specific health impacts of different sedentary postures (Korshøj et al. 2018). Previous research on occupational sedentary behaviour has suggested that non‐neutral sitting postures may be associated with LBP (Burdorf et al. 1993). However, these studies primarily focused on total ST in occupational settings, limiting the ability to assess the impact of different sitting postures on LBP. Some studies have suggested that short‐term exposure to certain sitting postures can induce LBP, while long‐term exposure to the same postures may yield different outcomes, highlighting the need for further research (Min et al. 2024).

Therefore, this study aims to examine the association between different types of sedentary behaviour and LBP risk. Additionally, it seeks to explore how increased time spent in specific sedentary behaviours affects lumbar disability index scores and LBP severity.

## 2 Methods

### 2.1 Study Participants

This study utilised data from the Korean Genome and Epidemiology Study (KoGES), a large‐scale cohort study. The study population consisted of adults aged 49–79 residing in Ansan, South Korea, who participated in the 2011–2012 KoGES survey aimed at preventing chronic disease among Koreans. A total of 3052 participants were recruited through telephone interviews, mail surveys, and home visits. After excluding 310 individuals with missing data on variables affecting LBP and sedentary behaviour (including sex, age, sleep duration, smoking status, alcohol consumption, income level, occupation type, obesity status, inflammation levels, and leisure‐time physical activity levels), 2742 participants were included in the final analysis. This study was approved by the Institutional Review Boards of Korea University Ansan Hospital and Seoul National University (IRB No. E2501/004‐006). Written informed consent was obtained from all participants after they were provided with a detailed explanation of the study’s purpose and procedures.

### 2.2 Measurement Variables

#### 2.2.1 LBP Risk

LBP was assessed using the Korean version of the Oswestry Disability Index (ODI). The ODI consists of 10 domains: pain intensity, personal care, lifting, walking, sitting, standing, sleeping, sexual activity, social life, and travelling. The total ODI score was calculated by summing the scores of all items, dividing the sum by the maximum possible score, and then multiplying by 100 to obtain a percentage score (Brokelman et al. 2012). The ODI is a well‐established tool for evaluating lumbar disability, with excellent test‐retest reliability (0.93) and internal consistency (0.92). LBP intensity was measured using the visual analogue scale (VAS), in which participants were asked to indicate their pain level on a 10‐cm scale, with 0 representing no pain and 10 indicating the most severe pain. The VAS has been reported to have very high test‐retest reliability (0.95) (Brokelman et al. 2012). LBP classification was based on previous studies. Participants who reported experiencing severe LBP in the past six months, had an ODI score of 20% or higher (Williams and Johnson 2024), or a VAS score of 4 or higher (Brokelman et al. 2012), were classified as the ‘LBP’ group. Those who did not meet these criteria were classified as the ‘Non‐LBP’ group.

#### 2.2.2 Types of Sedentary Behaviours

Total ST was calculated based on responses to the following questions: ‘On a typical weekday over the past week, how many hours did you spend sitting?’ and ‘On a typical weekend day over the past week, how many hours did you spend sitting?’ The daily total ST was determined using the formula: ((weekday ST × 5) + (weekend ST × 2))/7. Following previous studies, ST was categorised into two groups: ‘< 7 h’ and ‘≥ 7 h’ (Chau et al. 2013). To measure ST by type, participants answered detailed questions regarding the time spent on specific activities, including ‘watching TV (TV‐ST)’, ‘squatting (Squat‐ST)’, and ‘sitting on the floor (Floor‐ST)’. Based on previous findings indicating that sedentary behaviour exceeding two hours is associated with an increased risk of LBP, each category was classified into three groups: ‘< 2 h’, ‘2–4 h’, and ‘≥ 4 h’ (Macfarlane et al. 1997).

#### 2.2.3 Covariates

Data collection was conducted through face‐to‐face interviews with trained surveyors. Surveys were reviewed and revised for completeness on the same day. Height (cm) and weight (kg) were each measured once. The average of the three measurements was used in the analysis. Body mass index (BMI) was calculated as weight (kg) divided by height squared (m^2^). Alcohol consumption was asked, ‘Have you never consumed alcohol or have you abstained from alcohol from the beginning?’ Those who responded ‘yes’ were classified as ‘never drinkers’. Those who responded ‘no’ were asked an additional question: ‘Do you still drink alcohol?’ Participants who answered ‘no’ were classified as ‘former drinkers’, and those who answered ‘yes’ were classified as ‘current drinkers’. Smoking status was asked, ‘Have you ever smoked at least five packs (100 cigarettes) in your lifetime?’ Those who answered ‘no’ were categorised as ‘never smokers’. Those who answered ‘yes’ were asked a follow‐up question: ‘Do you currently smoke?’ Participants who answered ‘no’ were classified as ‘former smokers’, and those who answered ‘yes’ were classified as ‘current smokers’. Sleep Duration reported their average sleep duration on weekdays and weekends by answering the following questions: ‘How many hours do you typically sleep at night on weekdays?’ and ‘How many hours do you typically sleep at night on weekends over the past month?’ Daily sleep duration was calculated using the formula: ((weekday sleep duration × 5) + (weekend sleep duration × 2))/7 and was used as a continuous variable in the analysis. Household Income Level was assessed by asking, ‘What is your household’s average monthly income?’ Responses were categorised into the following income groups: ‘< 1,000,000 KRW’ (participants who answered < 500,000 KRW or 500,000–999,999 KRW), ‘1,000,000–2,000,000 KRW’ (participants who answered 1,000,000–1,499,999 KRW or 1,500,000–1,999,999 KRW), ‘2,000,000–3,000,000 KRW’ (participants who answered 2,000,000–2,999,999 KRW), ‘3,000,000–4,000,000 KRW’ (participants who answered 3,000,000–3,999,999 KRW), and ‘≥ 4,000,000 KRW’ (participants who answered 4,000,000–5,999,999 KRW or ≥ 6,000,000 KRW). Occupational categories were asked, ‘What is your current occupation? (14‐category classification)’. Responses were categorised into the following occupational groups: ‘Professional/Managerial/Clerical’ (professional, managerial, or clerical workers), ‘Sales/Service’ (salespersons or service workers), ‘Agricultural/Fishery/Technical’ (agriculture, forestry, fishery, hunting, or technical workers), ‘Homemakers’ (homemakers or domestic workers), and ‘Others’ (participants who reported other occupations). High‐sensitivity C‐reactive protein (hs‐CRP) levels were analysed using blood samples collected after an eight‐hour fast. Blood samples were processed onsite using a centrifuge and sent to the Seoul Clinical Laboratory, where hs‐CRP levels were measured using the ADVIA 1800 auto analyser (Siemens, USA). Participants' LTPA levels were assessed based on their responses regarding participation in vigorous‐intensity, moderate‐intensity, and walking activities over the past seven days. The total weekly leisure‐time physical activity (minutes per week) was calculated by summing the time spent in these activities. Based on previous research, participants were classified into two groups: ‘< 150 min/wk’ and ‘≥ 150 min/wk’ (Cho et al. 2021).

### 2.3 Statistical Analysis

All statistical analyses were conducted using STATA/IC 14.1 (STATA Corp., College Station, TX, USA). The demographic characteristics of the study population were analysed using descriptive statistics, including chi‐square tests for categorical variables and mean calculations for continuous variables. The results were expressed as percentages or as means with standard deviations (Tables 1 and 2).

**TABLE 1:** Baseline characteristics of the subjects by LBP status.

Characteristics of risk factors	Total (*n* = 2742)			
	Total	Non‐LBP	LBP	*p*‐value
	(*n* = 2742)	(*n* = 1571)	(*n* = 1171)	
Age (yr)	57.84 ± 7.01	58.32 ± 7.20	57.20 ± 6.70	< 0.001
Male (%)	53.17	57.10	47.91	< 0.001
ODI score	12.56 ± 14.84	2.65 ± 6.45	25.86 ± 12.30	< 0.001
VAS score	2.12 ± 2.74	0.32 ± 0.81	4.53 ± 2.57	< 0.001
BMI (kg/m^2^)	24.74 ± 2.92	24.69 ± 2.87	24.80 ± 3.00	0.037
Lean body mass (kg)	44.63 ± 8.19	44.88 ± 7.99	44.29 ± 8.44	0.059
hs‐CRP (mg/dL)	1.42 ± 3.38	1.48 ± 3.64	1.34 ± 2.99	0.259
Current drinking (%)	48.87	48.70	49.10	0.066
Current smoking (%)	13.49	14.00	12.81	0.034
Low income (%)	10.98	10.95	11.02	0.360
Sleep time (hr/day)	6.14 ± 1.19	6.22 ± 1.13	6.03 ± 1.26	< 0.001
Leisure PA (min/wk)	245.98 ± 299.18	261.77 ± 301.16	224.80 ± 295.30	0.001
Daily ST (hr/day)	5.75 ± 2.64	5.84 ± 2.63	5.63 ± 2.66	0.041
TV‐ST (hr/day)	2.66 ± 1.56	2.57 ± 1.55	2.77 ± 1.57	0.001
Squat‐ST (hr/day)	0.39 ± 0.86	0.28 ± 0.68	0.53 ± 1.04	< 0.001
Floor‐ST (hr/day)	1.44 ± 1.84	1.33 ± 1.76	1.58 ± 1.93	< 0.001
Classification of occupations (%)				
Managers, professionals, clerical worker	28.48	29.15	27.58	< 0.001
Services and sales worker	10.43	10.82	9.91	
Agricultural, forestry, fishery worker and technicians	15.68	16.23	14.94	
Homemaker	30.42	26.61	35.53	
Et cetera	14.99	17.19	12.04	

*Note:* Continuous data are presented as means (SD) and categorical data are presented as %.

Abbreviations: BMI, body mass index; hs‐CRP, high sensitivity‐C reactive protein; LBP, low back pain; ODI, oswestry disability index; PA, physical activity; VAS, visual analogue scale.

**TABLE 2:** Baseline characteristics of the subjects by different types of ST.

Characteristics of risk factors	Total (*n* = 2742)								
	TV‐ST (hr/day)			Squat‐ST (hr/day)			Floor‐ST (hr/day)		
	< 2 (*n* = 634)	≥ 2 (*n* = 2108)	*p*	< 2 (*n* = 2749)	≥ 2 (*n* = 222)	*p*	< 2 (*n* = 1892)	≥ 2 (*n* = 1079)	*p*
ODI score	11.10 ± 14.35	13.00 ± 14.96	0.005	12.07 ± 14.66	18.65 ± 15.71	< 0.001	12.44 ± 14.81	12.78 ± 14.89	0.568
VAS score	1.90 ± 2.63	2.18 ± 2.77	0.024	2.06 ± 2.72	2.75 ± 2.93	0.001	2.04 ± 2.73	2.25 ± 2.75	0.050
BMI (kg/m^2^)	24.51 ± 2.79	24.81 ± 2.96	0.024	24.78 ± 2.92	24.25 ± 2.94	0.014	24.71 ± 2.89	24.78 ± 2.99	0.524
Lean body mass (kg)	45.65 ± 8.07	44.32 ± 8.20	< 0.001	44.82 ± 8.19	42.27 ± 7.75	< 0.001	45.28 ± 8.22	43.47 ± 8.00	< 0.001
hs‐CRP (mg/dL)	1.49 ± 4.64	1.40 ± 2.90	0.588	1.45 ± 3.48	1.11 ± 1.67	0.173	1.45 ± 3.62	1.37 ± 2.90	0.546
Sleep time (hr/day)	6.19 ± 1.18	6.12 ± 1.19	0.189	6.13 ± 1.19	6.19 ± 1.21	0.530	6.16 ± 1.20	6.11 ± 1.17	0.299
Leisure PA (min/wk)	264.21 ± 342.22	240.50 ± 284.83	0.080	249.72 ± 304.75	199.68 ± 213.99	0.021	250.19 ± 304.16	238.47 ± 290.08	0.325
Daily ST (hr/day)	5.32 ± 2.93	5.88 ± 2.54	< 0.001	5.80 ± 2.66	5.12 ± 2.29	< 0.001	5.63 ± 2.70	5.96 ± 2.53	0.002

*Note:* Continuous data are presented as means (SD) and categorical data are presented as %.

Abbreviations: BMI, body mass index; hs‐CRP, high sensitivity‐C reactive protein; ODI, oswestry disability index; PA, physical activity; ST, sitting time; VAS, visual analogue scale.

To examine the association between sedentary behaviour types and the risk of low back pain (LBP), logistic regression analysis was performed to calculate odds ratios (OR) and 95% confidence intervals (CI) (Table 3). Additionally, to assess whether leisure‐time physical activity levels mediated the relationship between sedentary behaviour types and LBP risk, two separate models were employed.

**TABLE 3:** Odds ratio of LBP risk according to ST.

Characteristics of risk factors	Age‐sex adjusted (*n* = 2742)	Model 1 (*n* = 2742)	Model 2 (*n* = 2742)
Daily ST			
< 7 h/day	1.00 (reference)	1.00 (reference)	1.00 (reference)
≥ 7 h/day	0.93 (0.79, 1.10)	0.89 (0.76, 1.06)	0.89 (0.75, 1.05)
*p*‐trend	0.427	0.240	0.183
TV‐ST			
< 2 h/day	1.00 (reference)	1.00 (reference)	1.00 (reference)
2–4 h/day	1.25* (1.03, 1.52)	1.23* (1.01, 1.50)	1.23* (1.01, 1.50)
≥ 4 h/day	1.52*** (1.21, 1.90)	1.43** (1.14, 1.81)	1.44** (1.14, 1.82)
*p*‐trend	< 0.001	0.002	0.002
Squat‐ST			
< 2 h/day	1.00 (reference)	1.00 (reference)	1.00 (reference)
2–4 h/day	1.83*** (1.33, 2.52)	1.85*** (1.34, 2.56)	1.85*** (1.33, 2.56)
≥ 4 h/day	3.74*** (1.80, 7.80)	4.12*** (1.96, 8.66)	4.14*** (1.96, 8.72)
*p*‐trend	< 0.001	< 0.001	< 0.001
Floor‐ST			
< 2 h/day	1.00 (reference)	1.00 (reference)	1.00 (reference)
2–4 h/day	1.06 (0.88, 1.28)	1.07 (0.89,1.29)	1.07 (0.89, 1.29)
≥ 4 h/day	1.19 (0.95, 1.51)	1.18 (0.93, 1.49)	1.16 (0.92, 1.48)
*p*‐trend	0.121	0.181	0.205

*Note:* Model 1 adjusted age, sex, income level, sleep duration, alcohol consumption, current smoking, hs‐CRP, classification of occupations, daily ST, lean body mass. Model 2 adjusted model1 plus weekly leisure PA.

Abbreviations: BMI, body mass index; hs‐CRP, high sensitivity‐C reactive protein; LBP, low back pain; ODI, oswestry disability index; ST, sitting time; VAS, visual analogue scale.

^*^ *p* < 0.05.

^**^ *p* < 0.01.

^***^ *p* < 0.001.

Furthermore, to analyse changes in the ODI score and LBP intensity (VAS) according to increased sedentary behaviour duration, Multiple linear regression analysis was conducted, and *β* coefficients with 95% CI were reported (Table 4). Potential confounders that could influence both sedentary behaviour and LBP—such as age, sex, sleep duration, high‐sensitivity C‐reactive protein (hs‐CRP) levels, alcohol consumption, smoking status, income level, occupational type, obesity status, and leisure‐time physical activity—were adjusted for in the analyses.

**TABLE 4:** Relationship between ST posture and LBP‐VAS score and LBP‐ODI score according to daily ST level.

Characteristics of risk factors	All (*n* = 2742)			Daily ST					
				< 7 h/day (*n* = 1855)			≥ 7 h/day (*n* = 887)		
	*β*	SE	*p*	*β*	SE	*p*	*β*	SE	*p*
LBP‐VAS									
TV‐ST (hr/day)	0.01	0.03	0.801	0.03	0.04	0.470	−0.04	0.05	0.399
Squat‐ST (hr/day)	0.29	0.06	< 0.001	0.25	0.06	< 0.001	0.40	0.11	0.001
Floor‐ST (hr/day)	0.03	0.02	0.85	0.01	0.04	0.785	0.04	0.03	0.258
LBP‐ODI									
TV‐ST (hr/day)	0.55	0.18	0.003	0.71	0.23	0.002	0.16	0.31	0.597
Squat‐ST (hr/day)	2.72	0.32	< 0.001	2.60	0.37	< 0.001	3.20	0.64	< 0.001
Floor‐ST (hr/day)	0.26	0.15	0.091	0.24	0.22	0.279	0.22	0.21	0.284

*Note:* The regression *β* coefficient (*β*) with its standard error (SE) and the *p* value are given for each association. Multiple linear regression models adjusted age, sex, income level, sleep duration, alcohol consumption, current smoking, hs‐CRP, weekly leisure PA, classification of occupations, daily ST, lean body mass.

Abbreviations: BMI, body mass index; hs‐CRP, high sensitivity‐C reactive protein; LBP, low back pain, PA, physical activity; ST, sitting time.

## 3 Results

The demographic characteristics of the study population are presented in Table 1. Compared to the Non‐LBP group with less than 2 h of ST, the LBP group had higher age, male ratio, smoking rate, sleep duration, and leisure‐time physical activity participation time, while their total daily ST was lower. Additionally, the LBP group exhibited higher ODI scores, VAS scores, BMI, TV‐ST, Squat‐ST, Floor‐ST, and a higher proportion of homemakers.

Furthermore, the demographic characteristics associated with LBP by sedentary behaviour type are presented in Table 2. Across all types of sedentary behaviour, individuals who engaged in more than 2 h of sedentary behaviour had significantly lower lean body mass and higher total daily ST compared with those in the less than 2‐h group. Notably, TV‐ST and Squat‐ST, unlike Floor‐ST, showed significant differences in ODI scores, VAS scores, and BMI. Additionally, a significant difference in leisure‐time physical activity participation time was observed only in the Squat‐ST group between those who engaged for more than 2 h and those who did so for less than 2 h.

The independent associations between sedentary behaviour type and LBP risk are presented in Table 3. Total daily ST and Floor‐ST were not significantly associated with the risk of metabolic syndrome in any of the analytical models. However, TV‐ST and Squat‐ST showed significant associations across all models. After adjusting for all confounding variables, individuals who watched TV for 2–4 h and those who watched for more than 4 h had a 1.23‐fold (OR = 1.23, 95% CI = 1.01–1.50) and 1.44‐fold (OR = 1.44, 95% CI = 1.14–1.82) increased LBP risk, respectively, compared with those who watched TV for less than 2 h. Furthermore, watching TV for more than 2 h was statistically significantly associated with increased LBP risk (*p*‐trend = 0.002). Similarly, for Squat‐ST, individuals who engaged in squatting for 2–4 h and more than 4 h had a 1.85‐fold (OR = 1.83, 95% CI = 1.33–2.56) and 4.14‐fold (OR = 4.14, 95% CI = 1.96–8.72) increased LBP risk, respectively, compared to those who squatted for less than 2 h. The increase in LBP risk with more than 2 h of squatting was also statistically significant (*p*‐trend< 0.001).

The associations between sedentary behaviour type and LBP‐VAS and ODI scores are presented in Table 4. For VAS scores, each additional hour of squatting per day was associated with a 0.29‐point increase in pain intensity (*p* < 0.001), a trend observed in both the < 7‐h group (*β* = 0.25, *p* < 0.001) and the ≥ 7‐h group (*β* = 0.40, *p* < 0.001). Regarding ODI scores, each additional hour of squatting per day was associated with an increase of 0.57 points (*p* = 0.002) and 2.73 points (*p* < 0.001) in back pain intensity. This trend remained significant in the < 7‐h group (TV‐ST: *β* = 0.72, *p* = 0.002; Squat‐ST: *β* = 2.61, *p* < 0.001). However, in the ≥ 7‐h group, only squatting showed a positive association with increased back pain intensity (*β* = 3.19, *p* < 0.001).

## 4 Discussion

This study found that participants who engaged in more than two hours of TV‐ST and Squat‐ST exhibited a significantly higher risk of low back pain (LBP) compared with those who participated in less than two hours of TV‐ST, even after adjusting for various confounding factors. Additionally, LBP intensity was associated only with Squat‐ST, showing a positive correlation between Squat‐ST duration and VAS scores, regardless of total daily ST. Regarding the ODI score, TV‐ST was positively associated with ODI scores in the overall sample and among individuals with less than seven hours of daily ST, whereas Squat‐ST showed a positive association with ODI scores across all groups.

Previous research on LBP and sedentary behaviour has indicated that common sitting postures include floor sitting and squatting (Lui et al. 2018), which require prolonged upper‐body flexion, potentially contributing to LBP development. Studies have suggested that while total ST alone may not be directly associated with LBP (Solomonow et al. 2003), specific postures and prolonged awkward sitting positions in occupational settings significantly increase the risk (Hartvigsen et al. 2000; Lis et al. 2007). For instance, one study reported that prolonged sitting in an awkward posture at work increased the risk of LBP by 4.56 times (OR = 4.56, 95% CI = 2.59–8.03).

Additionally, helicopter pilots had a ninefold higher risk of developing LBP compared to non‐pilots (OR = 9.0, 90% CI = 4.9–16.4) (Lis et al. 2007). These findings suggest that excessive lumbar lordosis induced by certain sitting postures may increase spinal loading, leading to muscle stiffness and, ultimately, LBP (Min et al. 2024). Furthermore, lumbar muscle atrophy and spinal degeneration due to repetitive use have been identified as key contributors to LBP, though spinal degeneration appears to have a weaker association with LBP intensity (Simon and Hicks 2018).

The independent association of TV‐ST and Squat‐ST with increased LBP risk, even after adjusting for leisure‐time physical activity, may be attributed to their link with obesity (Table 3). In this study, the BMI of participants in the LBP group was significantly higher than that of the non‐LBP group (*p* = 0.037). Notably, unlike floor sitting (Floor‐ST), both TV‐ST (*p* = 0.024) and Squat‐ST (*p* = 0.014) groups showed significant BMI differences between those with more than two hours of engagement and those with less than two hours. These findings align with previous studies demonstrating the association between obesity and LBP (Ha 2011). Research suggests that obesity and overweight status impose excessive loads on spinal structures, increasing the likelihood of LBP (Ha 2011). Moreover, sedentary behaviour reduces energy expenditure, potentially contributing to weight gain (Saeidifard et al. 2018). Prolonged ST has also been linked to increased opportunities for food intake, leading to higher total energy consumption and an elevated risk of obesity and overweight status (Straker et al. 2005). Given these factors, TV‐ST and Squat‐ST may promote weight gain by reducing energy expenditure and increasing energy intake, ultimately exacerbating spinal loading and contributing to LBP (Ha 2011; Saeidifard et al. 2018; Straker et al. 2005).

The significantly higher OR for LBP risk, VAS scores, and ODI scores in Squat‐ST compared with other sedentary behaviours may be explained by the biomechanical effects of prolonged squatting(Tables 3 and 4). Squatting postures lead to relaxation of the erector spinae muscles, which play a crucial role in lumbar extension. This muscle relaxation may increase vulnerability to lumbar injury (Callaghan and Dunk 2002). Studies have shown that delayed activation of lumbar extensor muscles when transitioning from a squatting to a standing position can lead to spinal damage (Lui et al. 2018). Additionally, prolonged squatting in various occupational settings has been reported to increase LBP risk (Pal et al. 2015). Notably, participants who engaged in more than two hours of Squat‐ ST had significantly lower weekly leisure‐time physical activity levels (50.04 min/week less) than those with less than two hours of Squat‐ ST (Table 2); prior research has highlighted that regular physical activity supports lumbar function and aids in LBP rehabilitation. Consequently, a lack of physical activity may contribute to the development of LBP (Gordon and Bloxham 2016). Thus, insufficient physical activity (Gordon and Bloxham 2016) combined with delayed activation of lumbar extensor muscles may explain why Squat‐ST is particularly detrimental compared with other sedentary behaviours (Lui et al. 2018).

In contrast, Floor‐ST did not show a significant association with LBP risk, VAS scores, or ODI scores (Tables 3 and 4). Previous studies have suggested that in many Asian countries, where floor sitting is common, individuals often use cushions to enhance stability and reduce lumbar discomfort (Couzens et al. 2024). The lack of significant results for Floor‐ST in this study suggests the need for further research that considers variations in floor sitting conditions when assessing LBP risk.

Despite the strengths of this study, including its focus on the association between different sedentary behaviour types and LBP risk as well as LBP intensity and disability scores, some limitations must be acknowledged. First, sedentary behaviour duration and type were assessed through self‐reported questionnaires, which may introduce recall bias. Future studies should incorporate objective measures, such as accelerometer‐based ST tracking and detailed records of sedentary behaviour types, to improve data accuracy. Second, the cross‐sectional nature of this study limits the ability to establish causal relationships between sedentary behaviour and LBP. However, given that this study utilised national data with a large sample size, the findings serve as valuable baseline data for future longitudinal investigations.

## 5 Conclusion

This study identified prolonged TV‐ST and Squat‐ST as factors contributing to an increased risk of LBP and lumbar disability index. In particular, Squat‐ST was found to exacerbate the intensity of LBP. Maintaining awkward sitting postures can be as detrimental to health as prolonged sitting itself. Therefore, to mitigate chronic LBP, frequent posture changes and adequate rest should be encouraged during prolonged TV‐ST and Squat‐ST. Additionally, at both national and corporate levels, strategies to promote physical activity during leisure time should be implemented to strengthen and rehabilitate the lumbar muscles.

## Author Contributions

Study concept and design: DooYong Park and On Lee. Acquisition of data: DooYong Park. Analysis and interpretation of data: DooYong Park and On Lee. Draughting of the manuscript: DooYong Park and On Lee. Critical revision of the manuscript: DooYong Park and On Lee. Statistical analysis: DooYong Park. Administrative, technical, or material support: On Lee. Study supervision: On Lee.

## Ethics Statement

This study was approved by the Institutional Review Board (IRB) of Korea University Ansan Hospital and the Seoul National University Bioethics Committee (IRB No. E2501/004‐006). All participants (Sanghun Yim, DooYong Park) provided written informed consent prior to enrolment, and all study procedures were conducted in accordance with the Declaration of Helsinki and relevant local regulations.

## Conflicts of Interest

The authors declare no conflicts of interest.

## Data Availability

The data that support the findings of this study are available on request from the corresponding author. The data are not publicly available due to privacy or ethical restrictions.
